# A Cohort Study of the Effects of Daily-Diet Water-Soluble Dietary Fiber on Butyric Acid-Producing Gut Microbiota in Middle-Aged and Older Adults in a Rural Region

**DOI:** 10.3390/microorganisms10091813

**Published:** 2022-09-10

**Authors:** Satoshi Sato, Daisuke Chinda, Tadashi Shimoyama, Chikara Iino, Sae Kudo, Kaori Sawada, Tatsuya Mikami, Shigeyuki Nakaji, Hirotake Sakuraba, Shinsaku Fukuda

**Affiliations:** 1 Department of Gastroenterology and Hematology, Hirosaki University Graduate School of Medicine, Hirosaki 036-8562, Japan; 2 Division of Endoscopy, Hirosaki University Graduate School of Medicine, Hirosaki 036-8562, Japan; 3 Aomori General Health Examination Center, Aomori 030-0962, Japan; 4 Center of Healthy Aging Innovation, Hirosaki University Graduate School of Medicine, Hirosaki 036-8562, Japan

**Keywords:** gut microbiota, butyric acid-producing bacteria, water-soluble dietary fiber, daily food intake

## Abstract

Water-soluble dietary fiber is primarily a substrate for degradation of short chain fatty acids (SCFAs), particularly butyric acid, by gut microbiota. SCFAs have beneficial effects on the whole body. However, epidemiological studies on the association between water-soluble dietary fiber from daily food intake and butyric acid-producing bacteria are inconsistent. The purpose of this study was to determine the association between levels of water-soluble dietary fiber from daily food intake and gut microbiota, particularly butyric acid producers, in middle-aged and older adults in a rural area in Japan. We examined the effects of water-soluble dietary fiber intake on gut microbiota after adjusting for confounding factors. After propensity score matching, 520 subjects (260 in the low-intake group and 260 in the high-intake group) were selected. One year later after a follow-up survey, we re-classified the participants and again compared low- and high-intake groups. As a result, people with a high intake had a higher relative abundance of butyric acid-producing bacteria. It was also revealed that butyric acid-producing bacteria remained high in the group that maintained high intake the next year. We concluded that continuous intake of water-soluble dietary fiber from daily food is necessary to maintain sufficient amounts of butyric acid-producing bacteria.

## 1. Introduction

Dietary fiber is defined as digestion-resistant elements in foods that are not digested by human digestive enzymes [1]. For this reason, most dietary fibers can reach the colon and are fermented into short-chain fatty acids (SCFAs), such as butyric acid, propionic acid, and acetic acid [2,3,4]. SCFAs suppress the growth of harmful gut microbiota and promote intestinal peristalsis by lowering the pH in the intestine [5]. In addition, SCFAs not only protect the colon mucosa but also have beneficial effects on the whole body, such as anti-inflammatory effects and improvement of glucose metabolism, via the gut–brain axis and by increasing GLP-1 [6]. Epidemiological studies have demonstrated the protective effects of dietary fiber on ischemic heart disease, diabetes mellitus, and colon cancer, and these effects might be related to SCFA fermented by the gut microbiota [7,8].

Dietary fiber is classified as water-soluble and insoluble based on its solubility in the gastrointestinal tract. Water-soluble dietary fiber includes pectin, alginic acid, and mannan and is mainly used as a substrate for degradation of SCFAs by gut microbiota [9,10]. In contrast, water-insoluble dietary fibers, including cellulose, hemicellulose, and lignin, absorb water, increase stool volume, and stimulate peristalsis [11]. The Ministry of Health, Labor, and Welfare in Japan set the overall dietary fiber intake standard at 21 g for men and 18 g for women per day. However, despite the different effects of water-soluble and insoluble dietary fiber, no standard for the optimal intake of each kind of fiber has yet to be established. In particular, establishing effective dairy intake of water-soluble fiber, which is involved in SCFA production, may be important for disease prevention.

Studies on gut microbiota have expanded dramatically using next-generation sequencing for comprehensive analysis, and many studies have examined the association between gut microbiota and water-soluble dietary fiber administered as a prebiotic for a defined period of time [6,7,12,13]. Butyric acid is one of the short-chain fatty acids, which are important products of bacterial fermentation in the colon. Butyric acid is a major energy source of colon epithelium [14]. In addition, butyric acid has anti-inflammatory and osteogenic effects by inducing regulatory T cells [15,16]. Furthermore, butyric acid has been reported to have cancer-inhibiting effects via induction of apoptosis, inhibition of cell proliferation and angiogenesis [17]. Systematic reviews have reported that water-soluble dietary fiber increases *Bifidobacterium* and *Lactobacillus* [13]. *Bifidobacterium* and *Lactobacillus* are not capable of producing butyric acid, but they indirectly increase butyric acid-producing bacteria [13]. Butyric acid-producing bacteria are increased by the administration of water-soluble dietary fiber as a nutrient source [6,7]. Butyric acid production in the human body is assumed by many bacterial species belonging to the phylum *Firmicutes*, the class *Clostridia,* and the order *Clostridiales.* In particular, *Feacalibacterium* and *Roseburia* have been reported as representative butyric acid-producing bacteria [18]. However, epidemiological studies have been contradictory regarding the association between water-soluble dietary fiber ingested from daily food and butyric acid-producing bacteria [13]. Indeed, butyric acid-producing bacteria are greatly affected by various confounding factors other than water-soluble dietary fiber [13]. In addition, the gut microbiota varies with the type and amount of food ingested, as well as with age, sex, body size, smoking and drinking habits, and oral medications [19,20]. Therefore, different results have been obtained in previous epidemiological studies on gut microbiota [6,7,12]. Furthermore, most previous studies have been conducted using short-term, intensive administration of dietary fiber as a prebiotic. Few studies have examined the effects of dietary fiber from just daily food intake on gut microbiota [13]. Cross-sectional studies are insufficient to study such effects because dietary habits and the gut environment change over the course of a year, even among the same individuals. Therefore, cohort studies adjusted for the effects of these confounding factors are important when dealing with gut microbiota epidemiologically.

The purpose of this study was to determine the association between differences in water-soluble dietary fiber intake from daily food and gut microbiota in the general population of a rural area in Japan. We examined the changes in the effects of water-soluble dietary fiber intake on gut microbiota assemblages, especially butyric acid-producing bacteria, after adjusting for confounding factors.

## 2. Material and Methods

### 2.1. Study Subjects

**Figure 1:**
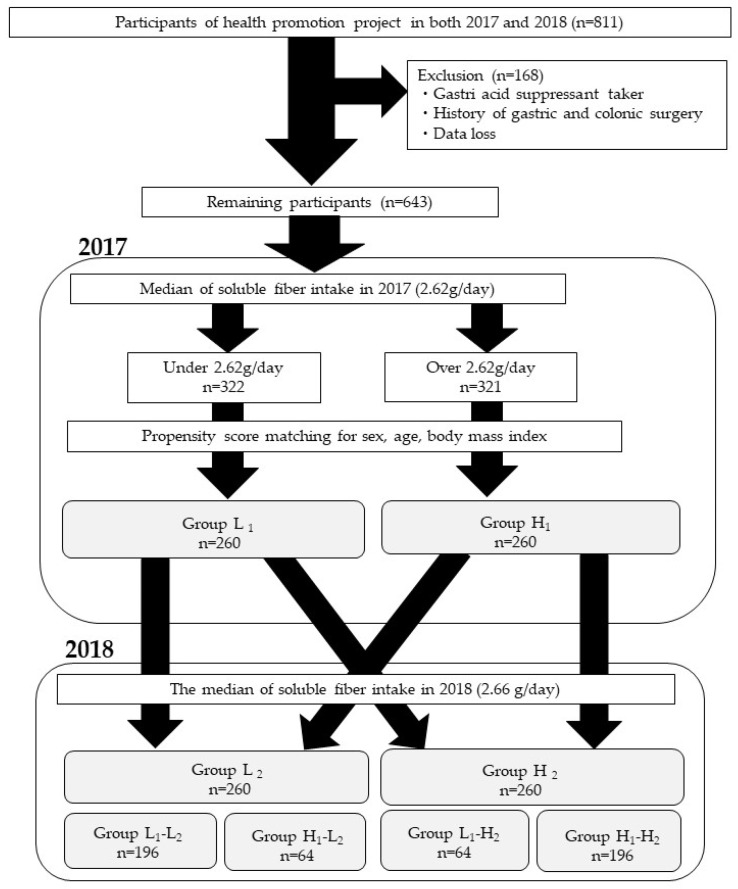
Study enrollment flow chart. Group L_1_: Water-soluble dietary fiber intake < 2.62 g/day in 2017; Group H_1_: Water-soluble dietary fiber intake ≥ 2.62 g/day in 2017; Group L_2_: Water-soluble dietary fiber intake < 2.66 g/day in 2018; Group H_2_: Water-soluble dietary fiber intake ≥ 2.66 g/day in 2018.

There were 811 adult participants in the Iwaki Health Promotion Projects held in June 2017 and 2018 in the Iwaki District of Hirosaki City located in northern Japan (Figure 1). Of these, 168 subjects who had either had a history of gastric or colonic surgery, were taking gastric acid secretion inhibitors, or had missing data were excluded. Subjects were divided into the low-intake group (322 subjects) or high-intake group (321 subjects), based on the median water-soluble dietary fiber intake (2.62 g/day) at the time of the 2017 survey. To equalize the background factors of both groups, propensity score matching was performed with sex, age, and BMI, all of which influence gut microbiota.

After propensity score matching, a total of 520 subjects, 260 in the low-intake group (Group L_1_) and 260 in the high-intake group (Group H_1_) were selected, and a follow-up survey was conducted (Figure 1). One year later, in 2018, the two groups were resorted using a median water-soluble dietary fiber intake of 2.66 g/day as the cut-off value for the low-intake group (Group L_2_) and the high-intake group (Group H_2_). Based on these results, the subjects were divided into four groups according to the change in water-soluble dietary fiber intake from 2017 to 2018: low- to low-intake group (L_1_-L_2_, 196 subjects), low to high-intake group (L_1_-H_2_, 64 subjects), high- to low-intake group (H_1_-L_2_, 64 subjects), and high- to high-intake group (H_1_-H_2_, 196 subjects).

The diversity of gut microbiota and the relative abundance of each bacterial species were compared between the low- and high-intake groups in 2017 and 2018. The bacterial species most commonly observed in the higher intake group in both 2017 and 2018 were defined as bacterial species associated with water-soluble dietary fiber. The changes in the relative abundance of water-soluble dietary fiber-associated bacterial species from 2017 to 2018 were then examined. In addition, the relative abundance of butyric acid-producing bacteria at the time of the 2018 survey was compared between the four groups.

### 2.2. Clinical Parameters

Water-soluble dietary fiber intake was calculated based on the results of the Brief Self-administered Diet History Questionnaire (BDHQ), a convenient diet assessment questionnaire developed in Japan. The BDHQ is a 4-page self-administered questionnaire that asks about the consumption frequency of selected foods to estimate the dietary intake of 58 commonly consumed food and beverage items in Japan for one month [21]. Subjects were given the BDHQ questionnaire in advance, and each subject was interviewed individually on the day of the project. Questionnaires were collected after confirming their answers. The following clinical parameters were recorded during the 2017 survey: sex, age, current medical history, previous medical history, medications, height, body weight, and body mass index. Diseases and medications added between 2017 and 2018 were recorded.

### 2.3. Next Generation Sequence Analysis of Gut Microbiota

Fecal samples were collected in commercial containers (TechnoSuruga Laboratory Co., Ltd., Shizuoka, Japan) and suspended in guanidine thiocyanate solution (100 mM Tris-HCL (pH 9.0), 40 mM Tris-EDTA (pH 8.0), 4M Guanidine Thiocyanate). These samples were kept at −80 °C prior to DNA extraction. According to previous studies, a series of representative bacterial species in the human gut microbiota were analyzed using primers for the V3–V4 region of 16S rDNA of prokaryotes [22]. Sequencing was performed using an Illumina MiSeq system (Illumina, San Diego, CA, USA). The methods for quality filtering of the sequences were as follows: the only reads that had quality value scores for scores ≥ 0 for more than 99% of the sequences were extracted for the analysis. Detection and identification of the bacteria from the sequences were performed using Metagenome@KIN software (R-4.1.1. World Fusion Co., Tokyo, Japan) and the TechnoSuruga Lab Microbial Identification database DB-BA 10.0 (TechnoSuruga Laboratory, Shizuoka, Japan) at 97% sequence similarity. Relative abundance is presented as the percent composition of reads for each bacterium relative to the total number of reads.

### 2.4. Statistical Analysis

Categorical variables are shown as frequencies, whereas continuous variables are shown as medians with interquartile ranges. Comparisons between the two groups were made using χ-square and Mann–Whitney U tests for independence, with the Wilcoxon signed rank test for dependency. Comparisons among the four groups were made using the Kruskal–Wallis test, followed by Steel–Dwass multiple comparisons. Spearman’s rank correlation coefficients were calculated to determine the correlation between the changes in water-soluble dietary fiber intake and butyric acid-producing bacteria. The family-wise error rate was adjusted using false discoveries. Microbiota were compared using linear discriminant analysis effect size (LEfse) [23].

Statistical analyses of the clinical data were performed using the Statistical Package for the Social Sciences (SPSS) version 28.0 (SPSS Inc., Chicago, IL, USA) and R software (R Foundation for Statistical Computing, version R-4.1.1). A *p*-value less than 0.05 was considered statistically significant.

### 2.5. Ethics Statement

This study was performed in accordance with the ethical standards of the Declaration of Helsinki and was approved by the ethics committee at Hirosaki University Medical Ethics Committee (authorization number: 2017-026 and 2018-062). All participants provided written informed consent.

## 3. Results

### 3.1. Participants’ Characteristics

The characteristics of the subjects are shown in Table 1. The high-intake group of water-soluble dietary fiber was older and had a higher BMI than the low-intake group.

The characteristics of Group L_1_ (260 subjects) and Group H_1_ (260 subjects) after propensity score matching with age, sex, and BMI are shown in Table 2. No significant differences in sex, age, or BMI were observed between the two groups. The median values of water-soluble dietary fiber intake at the time of the 2017 survey were 1.91 g/day for Group L_1_ and 3.30 g/day for Group H_1_. The characteristics of males and females for Group L_1_ and Group H_1_ are shown Table 3 and Table 4. In Group L_1_, males had higher BMI and lower intake of water-soluble dietary fiber intake than females. Contrarily, males had higher BMI and intake of total, water-soluble, and water-insoluble dietary fiber than females in Group H_1_.

### 3.2. Comparison of Gut Microbiota by Differential Intake of Water-Soluble Dietary Fiber in 2017

**Figure 2:**
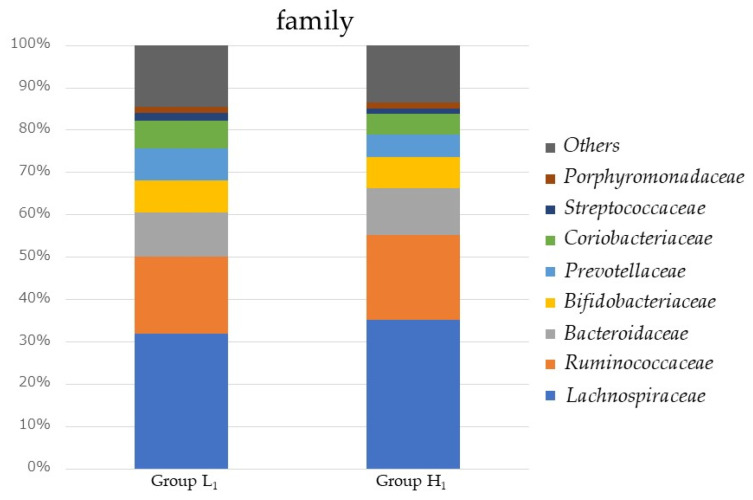
Comparison of the family relative abundance of gut microbiota in Group L_1_ and H_1_. Group L_1_: Water-soluble dietary fiber intake < 2.62 g/day in 2017; Group H_1_: Water-soluble dietary fiber intake ≥ 2.62 g/day in 2017.

**Figure 3:**
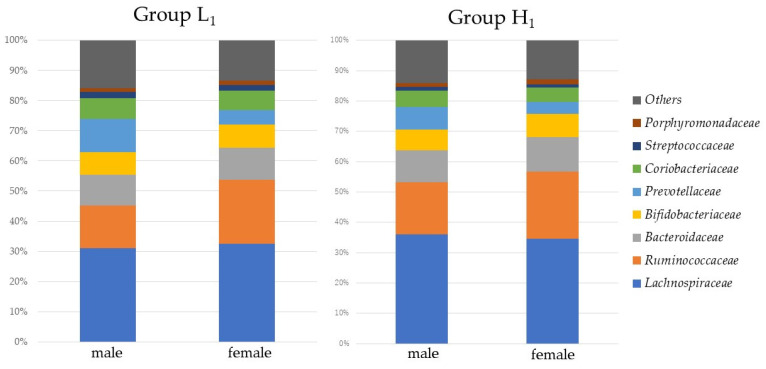
Comparison of the family relative abundance of gut microbiota in males and females for Group L_1_ and Group H_1_. Group L_1_: Water-soluble dietary fiber intake < 2.62 g/day in 2017; Group H_1_: Water-soluble dietary fiber intake ≥ 2.62 g/day in 2017.

Figure 2 shows a comparison of the relative abundance of gut microbiota in Groups L_1_ and H_1_. Group H_1_ showed significantly higher percentages of *Lachnospiraceae* and *Ruminococcaceae* (35.7% and 19.6%) than Group L_1_ (32.1% and 17.7%, *p*-values < 0.001 and 0.039, respectively). *Bifidobacteriaceae* was not significantly different at 5% relative abundance, and *Lactobacillaceae* was less than 0.01% of relative abundance in both groups. Figure 3 shows a comparison of relative abundance of gut microbiota in males and females for Group L_1_ and Group H_1_. In both Groups L_1_ and H_1_, males showed significantly lower percentages of *Ruminococcaceae* (14.0% and 17.2%) than females (21.2% and 22.0%, *p*-values < 0.001, respectively). In addition, males showed a significantly higher percentage of *Prevotellaceae* (11.8%) than females (4.8%, *p*-values < 0.001 and 0.039, respectively) in Group L1.

**Figure 4:**
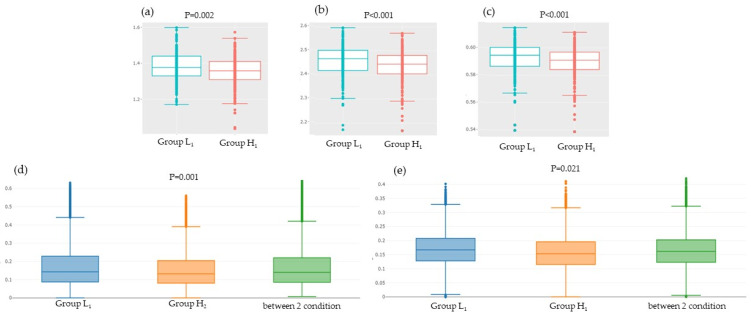
Comparison of the diversity of gut microbiota and water-soluble dietary fiber intake: (**a**) Shannon index; (**b**) Inverse Simpson; (**c**) Gini Simpson; (**d**) Weighted UniFrac distance; (**e**) Unweighted UniFrac distance. Group L_1_: Water-soluble dietary fiber intake < 2.62 g/day in 2017; Group H_1_: Water-soluble dietary fiber intake ≥ 2.62 g/day in 2017.

The Shannon index, inverse Simpson, and Gini Simpson indices, which show alpha diversity, were all lower in Group H_1_ than in Group L_1_ (Figure 4a–c). Both weighted and unweighted UniFrac distance, which represent beta diversity, showed significant differences between Groups L_1_ and H_1_ (Figure 4d,e).

### 3.3. Comparison of Water-Soluble Dietary Fiber Intake and Gut Microbiota in 2017 and 2018

**Figure 5:**
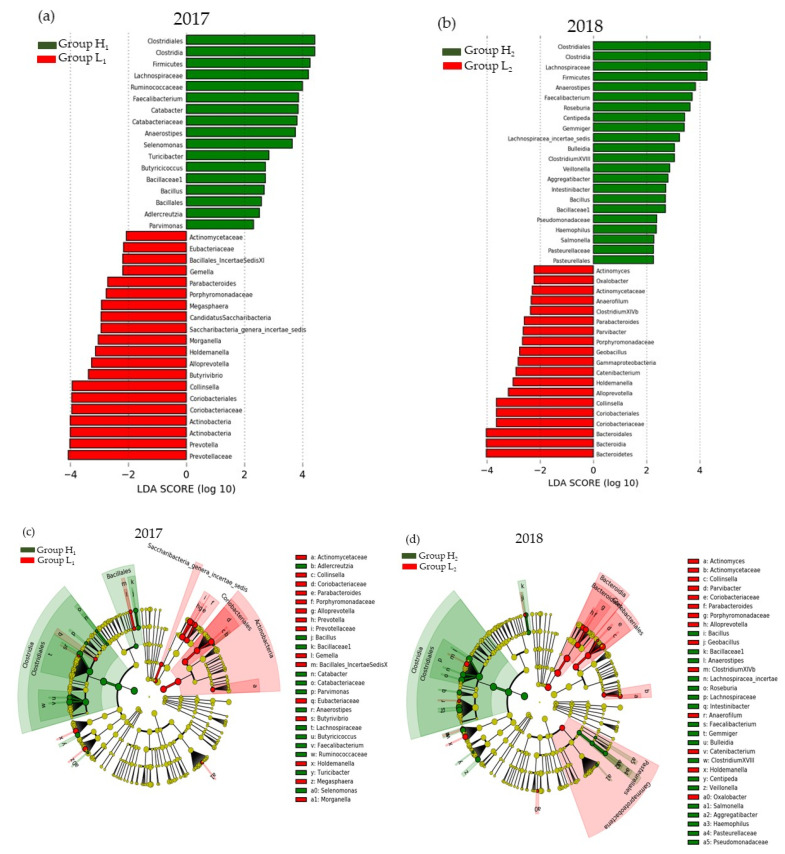
The LEfSe results of high- and low-intake groups of water-soluble dietary fiber: (**a**) The linear discriminant analysis in 2017; (**b**) The linear discriminant analysis in 2018; (**c**) The cladogram report in 2017; (**d**) The cladogram report 2018. Group L_1_: Water-soluble dietary fiber intake < 2.62 g/day in 2017; Group H_1_: Water-soluble dietary fiber intake ≥ 2.62 g/day in 2017; Group L_2_: Water-soluble dietary fiber intake < 2.66 g/day in 2018; Group H_2_: Water-soluble dietary fiber intake ≥ 2.66 g/day in 2018.

**Figure 6:**
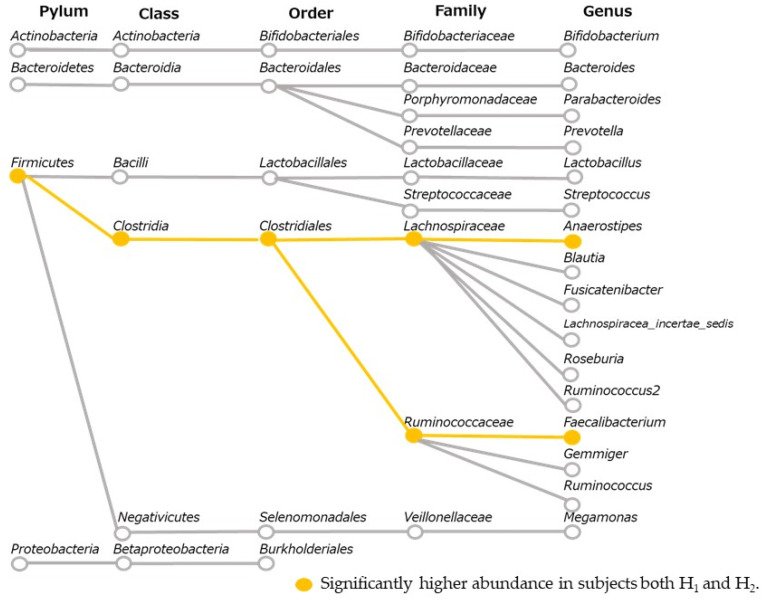
Systemic diagram of bacteria with more than 1% or more relative abundance in this study. Group H_1_: Water-soluble dietary fiber intake ≥ 2.62 g/day in 2017; Group H_2_: Water-soluble dietary fiber intake ≥ 2.66 g/day in 2018.

The LEfSe results of water-soluble dietary fiber intake and gut microbiota in 2017 and 2018 are shown in Figure 5. As the commonly detected bacteria in both years, the high-intake groups H_1_ and H_2_ had a significantly higher relative abundance of butyric acid-producing bacteria, *Anaerosipes* belonging to *Lachnospiraceae* and *Feacalibacterium* belonging to *Ruminococcaceae* (Figure 6). On the other hand, there were several bacteria with higher relative abundance in the low-intake Groups L_1_ and L_2_ in 2017 or 2018, respectively, but none were detected commonly in both years.

Although previous studies have reported that the administration of water-soluble dietary fiber as a prebiotic increased *Bifidobacterium* and *Lactobacillus* [24,25,26,27,28,29,30], there was no significant increase in this study.

### 3.4. Correlation between Changes in Water-Soluble Dietary Fiber and Relative Abundance of Butyric Acid-Producing Bacteria

Changes in water-soluble dietary fiber intake from 2017 to 2018 are shown in Table 5. There were no significant differences in the changes in water-soluble dietary fiber intake in L_1_-L_2_ and H_1_-H_2_. In contrast, L_1_-H_2_ and H_1_-L_2_ showed significant differences in water-soluble dietary fiber intake, although the changes were small, 0.81 g/day increase and 0.63 g/day decrease, respectively. H_1_-H_2_ had a higher intake than the other groups in both 2017 and 2018.

There was no significant correlation between changes in daily water-soluble dietary fiber intake and the relative abundance of butyric acid-producing bacteria from 2017 to 2018 in any of the four groups (Table 6).

### 3.5. Association of the Changes in Water-Soluble Dietary Fiber Intake and Relative Abundance of Butyric Acid-Producing Bacteria

**Figure 7:**
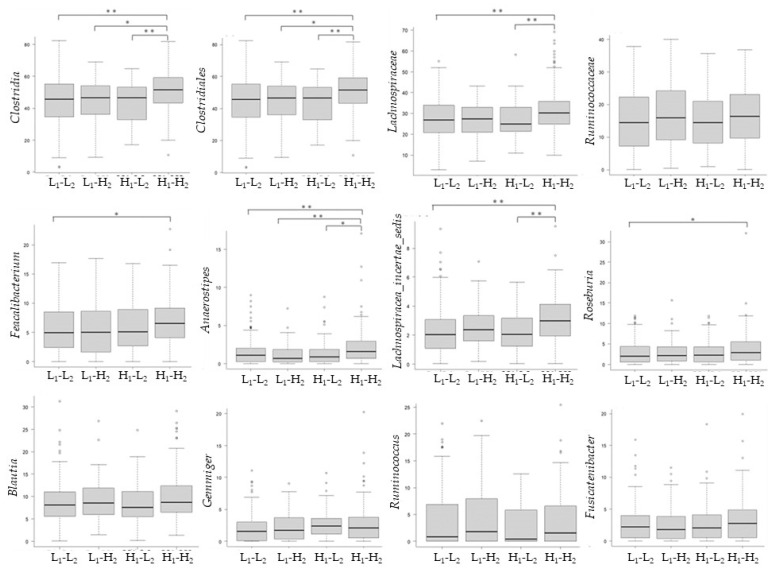
Comparison of butyric acid-producing bacteria among four groups classified by water-soluble dietary fiber intake from 2017 to 2018. L_1_-L_2_: Less than 2.62 g/day in 2017 and less than 2.66 g/day in 2018; L_1_-H_2_: Less than 2.62 g/day in 2017 and more than 2.66 g/day in 2018; H_1_-L_2_: More than 2.62 g/day in 2017 and less than 2.66 g/day in 2018; and H_1_-H_2_: More than 2.62 g/day in 2017 and more than 2.66 g/day in 2018. * < 0.05, ** < 0.01.

The comparison of butyric acid-producing bacteria among the four groups at the time of the 2018 survey revealed that H_1_-H_2_ had a significantly higher relative abundance of *Clostridia*, *Clostridiales*, *Lachnospiraceae*, *Anaerostipes*, *Feacalibacterium*, *Lachnospiraceae incertae sedis,* and *Roseburia* compared to the other groups (Figure 7).

## 4. Discussion

This is the first large cohort study to investigate the effects of water-soluble dietary fibers from daily food intake on gut microbiota in middle-aged and older adults. Our study revealed that individuals with a high intake of water-soluble dietary fiber had a higher relative abundance of butyric acid-producing bacteria. In addition, we revealed that butyric acid-producing bacteria remained high in the H_1_-H_2_ group with a continuously high intake for more than one year.

The Shannon, inverse Simpson, and Gini Simpson indices, which indicate diversity within an individual, were lower in the high water-soluble dietary fiber intake group in 2017 (Group H_1_). Most previous studies have reported that dietary fiber administration as a prebiotic does not change the diversity of the gut microbiota [31,32,33]. A comparison of the 2017 and 2018 surveys in this study revealed that the high water-soluble dietary fiber intake group (Group H_1_ and H_2_) had a higher relative abundance of bacteria belonging to the order *Clostridiales* in both years. On the other hand, in the low-intake group (Group L_1_ and L_2_), many bacteria increased in a single year, but no bacteria had a commonly high relative abundance in both 2017 and 2018. The reason for differences in α-diversity is that the high water-soluble dietary fiber intake group had a continuously high relative abundance of butyric acid-producing bacteria belonging to *Clostridiales*, whereas in the low-intake group, bacterial flora was unstable. The same reason could explain the significant difference in weight and unweighted UniFrac distance, which indicates diversity between individuals.

In this study, the bacterial species commonly detected in both 2017 and 2018 were defined as those associated with water-soluble dietary fiber. The groups with high water-soluble dietary fiber intake commonly had a higher relative abundance of *Anaerostipes* and *Feacalibacterium*. In laboratory studies, *Feacalibacterium* and *Roseburia* have been reported to produce butyric acid from water-soluble dietary fibers as substrates [18]. Therefore, higher water-soluble dietary fiber intake might increase butyric acid-producing bacteria. The relative abundance of *Anaerostipes* and *Feacalibacterium* was 3.78% and 7.25%, respectively. These values were higher than the average of 1.43% and 6.53% for a previously studied Japanese middle-aged group [34]. Previous studies have not observed an association between dietary fiber intake and intestinal butyric acid. However, our study targeted subjects with a high relative abundance of butyric acid-producing bacteria. Therefore, the association between dietary fiber and butyric acid-producing bacteria may be relatively greater.

In this study, the H_1_-H_2_ group who maintained high water-soluble dietary fiber intake over a year had significantly higher relative abundance of *Anaerostipes*, *Feacalibacterium*, *Roseburia,* and *Lachnospiraceae incertae sedis* at the time of the 2018 survey. In particular, a higher relative abundance was observed in H_1_-H_2_ than in L_1_-H_2_, whose water-soluble dietary fiber intake significantly increased from 2017 to 2018. In the H_1_-H_2_ group, the median of water-soluble dietary fiber intake was 3.47 g/day in 2017 and 3.54 g/day in 2018. These values were significantly higher than those of the other three groups. In 2018, the intake in the H_1_-H_2_ group (3.54 g/day) was significantly higher than that in the L_1_-H_2_ group (3.06 g/day). Continuous higher intake of water-soluble dietary fiber could be the reason for the high relative abundance of butyric acid-producing bacteria.

However, there was no significant correlation between the changes in water-soluble dietary fiber and the relative abundance of butyric acid-producing bacteria from 2017 to 2018 in any of the four groups. No significant correlations were observed in the L_1_-H_2_ and H_1_-L_2_ groups, whose water-soluble dietary fiber intake changed significantly over a year. Previous studies have reported that the administration of high doses of water-soluble dietary fiber as a prebiotic for a short-term increased butyric acid-producing bacteria [13]. However, in the present study, the L_1_-H_2_ and H_1_-L_2_ groups showed only small changes, albeit significant, in water-soluble dietary fiber intake of less than 1 g/day. The low levels of change in water-soluble dietary fiber intake might be the reason for the lack of significant correlations.

Previous studies have reported that the administration of water-soluble dietary fiber as a prebiotic increases *Bifidobacterium* and *Lactobacillus* [24,25,26,27,28,29,30]. However, no such association was observed in our study. *Bifidobacterium* are known to decrease with age [35]. Our study also observed a significant negative correlation between age and *Bifidobacterium* (correlation coefficient: −0.228 in 2017 and −0.162 in 2018). As the subjects in our study were mostly in the middle and elderly age groups, the association between water-soluble dietary fiber and *Bifidobacterium* was relatively small and showed a significant difference. In previous studies dealing with *Lactobacillus*, water-soluble dietary fiber was administered in high doses as a prebiotic to young people, and the age of subjects and methods of fiber intake were different from those in our study [28,29]. The fact that *Lactobacillus* was rarely present in our subjects (<0.001%) might also be the cause of the difference. In middle-aged and elderly people with a low relative abundance of *Bifidobacterium* and *Lactobacillus*, it would be difficult to increase these bacteria by water-soluble dietary fiber taken only from daily foods.

While our study was a cohort study with a large number of people over a one-year period, it still had several limitations. First, the participants in this study had a lower dietary fiber intake of approximately half of the national standard. Even in the high water-soluble dietary fiber intake groups, the subjects might not have consumed sufficient amounts. Second, the subjects of this study were middle-aged and elderly people in rural regions. It is well known that the gut microbiota changes with age and region, and it would not be appropriate to adapt the results of our study to younger people or urban residents. Third, although there were differences between males and females in both gut microbiota and water-soluble dietary fiber intake in this study, the effects of sex differences were not investigated in detail. In the future, it is necessary to clarify whether the differences in gut microbiota are due to differences in the intake of water-soluble dietary fiber or due to sex.

## 5. Conclusions

We revealed that the relative abundance of butyric acid-producing bacteria was higher with a higher intake of water-soluble dietary fiber from daily foods. In addition, because butyric acid-producing bacteria remained high when higher water-soluble dietary fiber intake was maintained, this suggests that the continuous intake of water-soluble dietary fiber is necessary to maintain sufficient amounts of butyric acid-producing bacteria.

## Figures and Tables

**Table 1:** Participants’ characteristics at baseline. Number or median (range).

	Low Intake (*n* = 322)	High Intake (*n* = 321)	*p*-Value
Males:Females	134:188	135:186	0.936
Age (years)	50.0 (38.0–60.8)	59.0 (45.0–67.0)	<0.001
BMI (kg/m^2^)	22.3 (20.2–24.3)	23.0 (20.5–25.3)	0.007
Total dietary fiber intake (g/day)	8.06 (6.40–9.54)	13.50 (11.90–16.00)	<0.001
Water-soluble dietary fiber intake (g/day)	1.93 (1.48–2.28)	3.40 (2.97–4.08)	<0.001
Water-insoluble dietary fiber intake (g/day)	5.93 (4.77–6.97)	9.58 (8.42–11.50)	<0.001

**Table 2:** Participants’ characteristics after matching for sex, age, and BMI.

	Group L_1_ (*n* = 260)	Group H_1_ (*n* = 260)	*p*-Value
Males:Females	112:148	107:153	0.722
Age (years)	53.0 (43.0–63.0)	55.0 (43.0–63.0)	0.847
BMI (kg/m^2^)	22.5 (20.9–24.8)	22.8 (20.4–25.0)	0.966
Total dietary fiber intake (g/day)	8.06 (6.36–9.60)	12.97 (11.60–15.54)	<0.001
Water-soluble dietary fiber intake (g/day)	1.91 (1.46–2.28)	3.30 (2.94–3.93)	<0.002
Water-insoluble dietary fiber intake (g/day)	5.95 (4.77–7.00)	9.30 (8.22–11.26)	<0.003

Number or median (range). Group L_1_: Water-soluble dietary fiber intake < 2.62 g/day in 2017. Group H_1_: Water-soluble dietary fiber intake ≥ 2.62 g/day in 2017.

**Table 3:** Participants’ characteristics of Group L_1_.

	Males (*n* = 107)	Females (*n* = 153)	*p*-Value
Age (years)	51.0 (42.0–61.0)	55.0 (47.0–63.0)	0.080
BMI (kg/m^2^)	23.3 (21.5–25.2)	22.1 (19.9–23.9)	<0.001
Total dietary fiber intake (g/day)	7.73 (6.34–9.49)	8.16 (6.52–9.66)	0.512
Water-soluble dietary fiber intake (g/day)	1.77 (1.39–2.25)	2.02 (1.49–2.30)	0.045
Water-insoluble dietary fiber intake (g/day)	5.65 (4.79–7.03)	6.34 (4.72–6.97)	0.985

Number or median (range). Group L_1_: Water-soluble dietary fiber intake < 2.62 g/day in 2017.

**Table 4:** Participants’ characteristics of Group H_1_.

	Males (*n* = 112)	Females (*n* = 148)	*p*-Value
Age (years)	52.0 (39.0–63.0)	57.0 (45.0–63.0)	0.092
BMI (kg/m^2^)	24.0 (22.1–26.1)	21.6 (19.3–23.8)	<0.001
Total dietary fiber intake (g/day)	14.18 (12.19–17.12)	12.55 (11.25–14.70)	<0.001
Water-soluble dietary fiber intake (g/day)	3.47 (3.07–4.28)	3.22 (2.86–3.77)	<0.001
Water-insoluble dietary fiber intake (g/day)	9.96 (8.60–12.33)	8.96 (8.09–10.45)	<0.001

Number or median (range). Group H_1_: Water-soluble dietary fiber intake ≥ 2.62 g/day in 2017.

**Table 5:** The change of water-soluble dietary fiber intake from 2017 to 2018.

	2017	2018	*p*-Value ^#^
L_1_-L_2_	1.80 (1.40–2.19)	1.83 (1.38–2.18)	0.462
L_1_-H_2_	2.25 (1.84–2.40)	3.06 (2.88–3.44)	<0.001
H_1_-L_2_	2.91 (2.79–3.17)	2.28 (1.92–2.50)	<0.001
H_1_-H_2_	3.47 (3.12–4.15)	3.54 (3.16–4.22)	0.446
*p*-value ^†^	<0.001	<0.001	

^†^: Steel–Dwass test; **^#^***:* Wilcoxon signed rank test median (range). L_1_-L_2_: less than 2.62 g/day in 2017 and less than 2.66 g/day in 2018. L_1_-H_2_: less than 2.62 g/day in 2017 and more than 2.66 g/day in 2018. H_1_-L_2_: more than 2.62 g/day in 2017 and less than 2.66 g/day in 2018. H_1_-H_2_: more than 2.62 g/day in 2017 and more than 2.66 g/day in 2018.

**Table 6:** Correlation of the amount of the change for one year between water-soluble dietary fiber intake and butyric acid-producing bacteria abundance.

	L_1_-L_2_		L_1_-H_2_		H_1_-L_2_		H_1_-H_2_	
	ρ	q	ρ	q	ρ	q	ρ	q
Class								
*Clostridia*	0.001	0.988	−0.020	0.983	0.162	0.839	−0.096	0.410
Order								
*Clostridiales*	0.001	0.988	−0.020	0.983	0.162	0.839	−0.096	0.410
Family								
*Lachnospiraceae*	0.074	0.988	0.040	0.983	0.031	0.950	−0.070	0.500
*Ruminococcaceae*	−0.051	0.988	−0.013	0.983	0.113	0.839	−0.107	0.410
Genus								
*Anaerostipes*	0.126	0.720	−0.081	0.983	0.124	0.839	−0.124	0.410
*Blautia*	0.018	0.988	−0.003	0.983	−0.008	0.950	−0.020	0.845
*Lachnospiracea incertae sedis*	−0.051	0.988	0.230	0.306	−0.029	0.950	−0.014	0.845
*Roseburia*	−0.025	0.988	0.238	0.306	−0.011	0.950	0.075	0.500
*Faecalibacterium*	−0.028	0.988	0.077	0.983	0.058	0.950	0.053	0.597

ρ: Spearman’s rank correlation coefficient. q-value: False Discovery Rate (Benjamini and Hochberg). L_1_-L_2_: less than 2.62 g/day in 2017 and less than 2.66 g/day in 2018. L_1_-H_2_: less than 2.62 g/day in 2017 and more than 2.66 g/day in 2018. H_1_-L_2_: more than 2.62 g/day in 2017 and less than 2.66 g/day in 2018. H_1_-H_2_: more than 2.62 g/day in 2017 and more than 2.66 g/day in 2018.

## Data Availability

The data presented in this study are available upon request from the corresponding author. The data were not publicly available because of privacy and ethical restrictions.
